# Age- and Sex-Specific Mortality Patterns in an Emerging Wildlife Epidemic: The Phocine Distemper in European Harbour Seals

**DOI:** 10.1371/journal.pone.0000887

**Published:** 2007-09-12

**Authors:** Tero Härkönen, Karin Harding, Thomas Dau Rasmussen, Jonas Teilmann, Rune Dietz

**Affiliations:** 1 Swedish Museum of Natural History, Stockholm, Sweden; 2 Department of Marine Ecology, Göteborg University, Göteborg, Sweden; 3 National Environmental Research Institute, Roskilde, Denmark; The Rockefeller University, United States of America

## Abstract

Analyses of the dynamics of diseases in wild populations typically assume all individuals to be identical. However, profound effects on the long-term impact on the host population can be expected if the disease has age and sex dependent dynamics. The Phocine Distemper Virus (PDV) caused two mass mortalities in European harbour seals in 1988 and in 2002. We show the mortality patterns were highly age specific on both occasions, where young of the year and adult (>4 yrs) animals suffered extremely high mortality, and sub-adult seals (1–3 yrs) of both sexes experienced low mortality. Consequently, genetic differences cannot have played a main role explaining why some seals survived and some did not in the study region, since parents had higher mortality levels than their progeny. Furthermore, there was a conspicuous absence of animals older than 14 years among the victims in 2002, which strongly indicates that the survivors from the previous disease outbreak in 1988 had acquired and maintained immunity to PDV. These specific mortality patterns imply that contact rates and susceptibility to the disease are strongly age and sex dependent variables, underlining the need for structured epidemic models for wildlife diseases. Detailed data can thus provide crucial information about a number of vital parameters such as functional herd immunity. One of many future challenges in understanding the epidemiology of the PDV and other wildlife diseases is to reveal how immune system responses differ among animals in different stages during their life cycle. The influence of such underlying mechanisms may also explain the limited evidence for abrupt disease thresholds in wild populations.

## Introduction

An array of new emerging diseases originating from wildlife reservoirs elucidates the need for more detailed epidemiological models to predict new outbreaks and facilitate their control [1]. However, owing to practical circumstances in sampling wild animal populations, vital information necessary to develop more pertinent models is mostly lacking. In particular, models of wildlife diseases are often compelled to ignore age and sex dependent features of the epidemiology, which is unfortunate since the pattern of age specific mortality can have a profound influence on the evolution of disease resistance [2], [3] and the rate of decline in herd immunity [3]. In this case study, we investigate the age and sex specific aspect of recurrent outbreaks in one of the best documented large-scale wildlife epidemics: the Phocine Distemper Virus (PDV) that caused two severe mass mortalities in the European metapopulation of harbour seals (*Phoca vitulina*) [4], [5], [6], [7]. More than 23,000 seals died in the 1988 epidemic, whereas the death toll exceeded 30,000 seals in the 2002 event. [7]. Mortality rates ranged between 22% and 66% in populations along mainland Europe, while British stocks were less affected [7]. The disease is enzootic in Arctic seals such as harp seals (*Pagophilus groenlandica*) [8], and the risk for repeated cross-species infection in the future is pending, since grey seals (*Halichoerus grypus*) could act as immune carriers in this process [7], [9], [10]. Mathematical modelling has shown that the risk for quasi extinction in harbour seals is substantially increased by repeated PDV outbreaks [11], where one major component affecting the mortality rate of repeated epidemics is the level of immunity amongst survivors [12], [13].

However, quantifying immunity and pathogen exposure in surviving seals has proven difficult. Serological studies following the 1988 epidemic gave estimates for the proportion of seals that might have escaped infection. Although these studies encompassed more than a thousand serum samples [4], [14], results were inconclusive, since it remains difficult to relate specific titre levels to PDV exposure to functional immunity [15]. Whilst individuals with titres over a certain threshold level have clearly been in contact with the virus or a similar virus such as CDV, individuals with lower titres cannot be excluded from having been exposed to the virus, nor do they necessarily lack innate immunity to the PDV [15]. A conclusive test on functional immunity is a repeated exposure to the same infective agent. The second outbreak of PDV in 2002 across mainland Europe provided a full scale natural experiment. The current study analyses the age- and sex-composition of the disease-related mortality in the two outbreaks and draws conclusions about disease dynamics and immunity.

## Results and Discussion

Males suffered significantly greater mortality (430 in 1988 and 1,142 in 2002) as compared with females (354 and 1,003) in both epidemics (Chi-square test p =  2.6^−7^), a difference that would be further enhanced if the fact that sex ratios in harbour seal populations are biased in favour of females (1∶0.88) had been taken into account. The skew in sex ratio in non epidemic conditions originates from a higher annual mortality of adult males [16].

The age structure of female and male seals that died during the 1988 epidemic deviated substantially from the estimated pre-epidemic stable age distributions. Adults (4–35 yrs) suffered substantially higher mortality rates than sub-adults (1–3 yrs) (Table 1). The age specific mortality rates were remarkably similar in 1988 and 2002 for seals up to 13 years of age. The age distributions deviated from the projected age distributions for both females (Kolmogorov-Smirnov two-sample test, p<0.01) and males (p<0.001). Again sub-adults suffered lower mortality than expected compared to adults in age classes 4–13 (Table 1). However, seals in age class 14 years and older showed substantially lower mortality rates in 2002 compared with 1988. In 1988 60 females (14+years) were found while 43 females were expected to be found (based on the estimated age distribution and sample size), and 48 males (14+years) were found compared to the 32 expected. Out of the total 1,003 females and 1,142 males collected in 2002 , 124 females and 92 males were expected to be 14 years or older. However, only four females and eight males were 14 years or older (Table 1). The number of older seals found in 2002 is therefore an order of magnitude lower than expected, and indistinguishable from the natural annual mortality, i.e. about 5% for adult females and 9% for adult males [17]. The absence of seals older than 14 years in the 2002 sample strongly indicates that all seals were exposed to PDV in 1988 and that the survivors developed complete immunity, protecting them from elevated mortality in 2002.

**Table 1:** Age categories of actually observed numbers (*N*) and expected numbers (*E* 1) of female (f) and male (m) seals that died in the 1988 and 2002 PDV epidemics.

Year	1988				2002			
Age group (Yr)	*N*_f_	*E*_f_	*N*_m_	*E*_m_	*N*_f_	*E*_f_	*N*_m_	*E*_m_
Juvenile (1–3)	**103**	141	**85**	194	**341**	424	**311**	544
Adults (4–13)	**191**	170	**297**	204	**657**	455	**822**	506
Adults (14)	**10**	6	**10**	6	**1**	0	**1**	0
Adults (>14)	**50**	37	**38**	26	**4**	124^2^	**8**	92^2^
Sum	**354**	354	**430**	430	**1003**	1003	**1142**	1142

**Figure 1:**
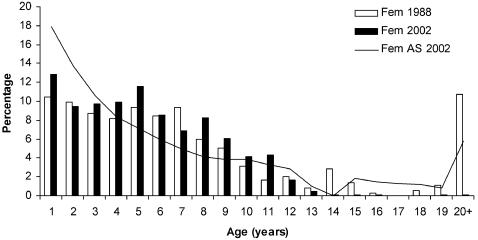
Age structures (%) of 354 females that died in the Skagerrak, Kattegat and the Baltic collected during the 1988 epidemic (white columns) compared with the age structure of the 1003 female seals collected in the Skagerrak, Kattegat , Baltic and the Danish Wadden Sea in 2002 (filled columns). The age structure (AS) just prior to the 2002 epidemic (line) is indicated.

**Figure 2:**
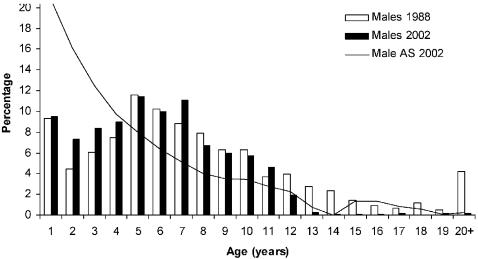
Age structures (%) of 430 males that died in 1988 (white columns) and of the 1142 males collected in 2002 (filled columns). The estimated age structure (AS) of total population in 2002 (line) is indicated.

1 Expected numbers based on the stable age distribution in 1988, and the projected age distribution in 2002 (Figs 1 and 2).

2 The 95% confidence interval (0.012) of the mean annual rate of increase (λ = 1.116) gives a range of expected numbers of 108–142 for females and 80–105 for males.

The data presented here indicate that PDV exposure leads to complete life-long immunity of survivors. Immunity of survivors has been shown to decrease the impact of repeated epizootics on population growth rates and to decrease the risk for quasi-extinction [13], [14]. On the other hand, acquired immunity can influence the selection pressure on innate disease resistant genotypes and can thereby maintain high levels of mortality during outbreaks for a longer time period than would be expected if only innate immunity had been present [3].

The low prevalence of PDV-specific anti-bodies in serological samples from Scottish harbour seals after 1988 [14] suggests the dynamics of the PDV could have been different in this region, a pattern repeated in 2002 when the disease barely reached epidemic levels [7]. Also in England, different dynamics are suggested by the substantially lower mortality rates in 2002, both compared with the 1988 epidemic, and populations along mainland Europe in 2002 [18].

We found that adults suffered substantially greater mortality rates compared with juveniles (1–3 years), both in 1988 and in 2002 (Figs 1 and 2). Since genetic differences are not to be expected among these age groups, this finding indicates that genetic differences cannot play an important role in explaining survival patterns in the study region. Rather, physiological differences co-varying with age and sex, governed the mortality patterns across mainland Europe. One of many future challenges in understanding the epidemiology of the PDV and other wildlife diseases is to discern how immune system responses differ among seals in different stages during their life cycle. The influence of such underlying mechanisms may explain the limited evidence for abrupt disease thresholds in wild populations.

## Materials and Methods

Among many other samples, canine teeth were collected from each sampled seal carcass during both mass mortalities. In 1988 teeth from 784 animals older than pups of the year were taken from the Danish and Swedish parts of the Skagerrak, the Kattegat, and the Baltic, whereas 1,953 teeth were taken from the same areas in 2002. In the latter year the material was supplemented with 192 teeth from seals older than pups from the Danish Wadden Sea, giving a total of 2,145 teeth (Table 1). Thus, samples were obtained from all major populations across mainland Europe. In 2002 we also collected 377 pups of the year (POY), out of which larger pups were aged using teeth, whereas seals shorter than 95 cm were regarded as pups of the year [17]. POY were excluded from comparisons of age specific mortality, due to their varying association to their mothers over the nursing period, and lower recovery rate of pup carcasses, but data indicate that close to 100% of the pups died in 1988 in severely affected regions [16].

Crowns of sampled teeth were removed, and the cementum of the roots were used for age determination [19]. Teeth from both epidemics were sectioned using a freezing microtome (Leica Cryostat CM 1510 and Frigomobil 1206) at the National Environmental Research Institute, Denmark. Central longitudinal 14µm thick sections were cut [19]. Before mounting on glass slides and being treated with 5% gelatine, the sections were kept in demineralised water (pH 8) for a minimum of 20 minutes. About 4–5 sections of each tooth were mounted on glass slides. The dried sections were stained with a 0.032% solution of Toluidine Blue 0 (“Certistan”, Merk, Germany), and dissolved in alkaline water (pH 8–9) for ten minutes. Excess stain was removed in two successive demineralised water baths. The sections were mounted permanently using Entellan, and cover clips. Using a reference material of animals with known age, teeth were then determined to age by counting the growth layer groups. A sub-sample of teeth was used in blind tests with three different readers [19].

To project age structure and population size from time *t* to *t+1*, a column vector (**n**), which includes the number of individuals in each age class, is multiplied by the population projection matrix (**L**). Thus, 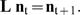
(1)The asymptotic population growth rate is given by the dominant eigenvalue of **L** and denoted λ_1_ and the stable age distribution is given by the right eigenvector [20].

Before 1988, the harbour seal population across mainland Europe had been increasing steadily by 12% per annum, which is close to the maximum growth capacity of harbour seals [21], [22], implying fertility rates close to 1 in sexually mature females, which also was confirmed by histological investigations of ovaries [17]. A well known fact is that after periods of exponential growth the age structure stabilises, and the expected numbers of individuals in each age class can be estimated from the life history matrix [20], [22], [23]. Given the observed annual rate of increase at 12%, it is readily seen that the deviation from the stable age distribution is negligible after 10 years [16], [24], [25] also in extreme scenarios. This is also confirmed by empirical studies, where proportions of pups remained constant in relation to the size and structure of the population [22]. Consequently, the possible minor deviations from assumed stable age distributions do not affect the results of this study.

To avoid sample biases, all carcasses washed ashore from the largest colonies were collected, which represented about 95% of all seals that died at those sites [16]. Further, the estimated age structure of the surviving population in 1988 was projected 20 years ahead, and the population growth rate was predicted to substantially exceed the maximum long-term growth rate of harbour seals (13% per annum). Later empirical studies showed that the population growth rate was close to 20% per annum during the years following the 1988 epidemic, which could be explained by that the population was initially dominated by young adult females [22].

The age structure of the seals killed in the 1988 epidemic [17] was used to estimate the age composition of the survivors in 1988 [16] (Figs 1 and 2). Constructing Leslie matrices for the female and male segments separately, and parameterised by the rate of annual population increase at 1.116 (95% confidence interval±0.012) over the period 1988 to 2000 [22], and age specific fertility and survival rates [16], we projected the population structure up to 2002 (Figs 1 and 2). The estimated pre-epidemic age composition in 2002 also represents the expected age structure of seals that died during the 2002 epidemic, if mortality rates in all age classes had been identical. We compare the age structure of the seals that died in 2002 to the estimated age composition of the total population in 2002 before the disease outbreak.
